# Eutectic Mixture Formation and Relaxation Dynamics of Coamorphous Mixtures of Two Benzodiazepine Drugs

**DOI:** 10.3390/pharmaceutics15010196

**Published:** 2023-01-05

**Authors:** Sofia Valenti, Claudio Cazorla, Michela Romanini, Josep Lluís Tamarit, Roberto Macovez

**Affiliations:** Grup de Caracterització de Materials, Departament de Física and Barcelona Research Center in Multiscale Science and Engineering, Universitat Politècnica de Catalunya, EEBE, Av. Eduard Maristany 10-14, E-08019 Barcelona, Spain

**Keywords:** valium, coamorphous formulations, dielectric spectroscopy, glass transition, ring-inversion, physical stability of glasses, heteromolecular interactions

## Abstract

The formation of coamorphous mixtures of pharmaceuticals is an interesting strategy to improve the solubility and bioavailability of drugs, while at the same time enhancing the kinetic stability of the resulting binary glass and allowing the simultaneous administration of two active principles. In this contribution, we describe kinetically stable amorphous binary mixtures of two commercial active pharmaceutical ingredients, diazepam and nordazepam, of which the latter, besides being administered as a drug on its own, is also the main active metabolite of the other in the human body. We report the eutectic equilibrium-phase diagram of the binary mixture, which is found to be characterized by an experimental eutectic composition of 0.18 molar fraction of nordazepam, with a eutectic melting point of *T*_e_ = 395.4 ± 1.2 K. The two compounds are barely miscible in the crystalline phase. The mechanically obtained mixtures were melted and supercooled to study the glass-transition and molecular-relaxation dynamics of amorphous mixtures at the corresponding concentration. The glass-transition temperature was always higher than room temperature and varied linearly with composition. The *T*_e_ was lower than the onset of thermal decomposition of either compound (pure nordazepam decomposes upon melting and pure diazepam well above its melting point), thus implying that the eutectic liquid and glass can be obtained without any degradation of the drugs. The eutectic glass was kinetically stable against crystallization for at least a few months. The relaxation processes of the amorphous mixtures were studied by dielectric spectroscopy, which provided evidence for a single structural (α) relaxation, a single Johari–Goldstein (β) relaxation, and a ring-inversion conformational relaxation of the diazepinic ring, occurring on the same timescale in both drugs. We further characterized both the binary mixtures and pure compounds by FTIR spectroscopy and first-principles density functional theory (DFT) simulations to analyze intermolecular interactions. The DFT calculations confirm the presence of strong attractive forces within the heteromolecular dimer, leading to large dimer interaction energies of the order of −0.1 eV.

## 1. Introduction

Glassy active pharmaceutical ingredients (APIs) have higher solubility and bioavailability than their crystalline counterparts, but they are in unstable states that are prone to transform into the more stable crystal phase. Binary or multicomponent amorphous systems of compounds that do not form cocrystals are, in some cases, less prone to recrystallize due to the kinetic barrier to diffusion preventing phase separation. This effect is exploited in amorphous polymer dispersions, for example, which have, however, the drawback that the drug content is relatively low and that, in the presence of macromolecules, the control of sample morphology and heterogeneity is less straightforward [1,2,3,4].

A recent study has shown that several amorphous binary API mixtures display the largest kinetic stability against recrystallization when the composition of the mixture is close to the eutectic one [5]; however, this result might not be general to all binary glass-forming liquid mixtures [6]. Here, we describe a strategy to obtain a kinetically stable amorphous binary system of two APIs in which one is the main active metabolite of the other in the human body. The two drugs under scrutiny are diazepam (commercially known under trademarks such as Valium) and the closely related nordazepam derivative (commercialized as Nordaz, Stilny, Madar, Vegesan, and Calmday).

The choice of APIs is further motivated by the fact that benzodiazepines constitute a large family of psychoactive drugs that are largely prescribed worldwide for their relative safety within therapeutic dosages, but that are generally only poorly soluble in water. The most known diazepam derivative, in fact, displays a solubility of 0.015 mmol L^−1^ at room temperature [7,8] and is classified either as a class I or class II drug of the biopharmaceutical classification system (BCS) depending on the dosage [9]. For improving the bioavailability of these related APIs, high dissolution and absorption rates (as achievable via an amorphous formulation) are important to achieve, in the case of diazepam, because they are mandatory, e.g., when it is used for rapid termination of febrile convulsions and epileptic seizures [10,11].

We established the extended (equilibrium and nonequilibrium) binary phase diagram of the system. The equilibrium-phase diagram is characterized by eutectic composition at a nordazepam molar fraction of 0.18 and eutectic melting point at 395 K, while the glass-transition temperature of the amorphous mixtures is found to scale linearly with composition. The amorphous eutectic mixture is shown to have much better kinetic stability than the pure compounds. The amorphous binary system is further characterized by dielectric spectroscopy to extract information about the characteristic time of the structural and secondary relaxation dynamics, as well as about the dielectric constant and ionic conductivity of the mixtures. Finally, we carried out first-principles density functional theory (DFT) calculations and FTIR spectroscopy measurements to analyze intermolecular interactions in the binary glass. The FTIR spectra suggest a predominance of ring–ring interactions compared to H-bonding. The DFT calculations confirm the presence of strong attractive forces without H-bonding within the heteromolecular dimer, rendering large formation energies of the order of −0.1 eV.

The formation of kinetically stable amorphous mixtures of two related compounds in which one is the metabolite of the other, as presented here, may allow researchers to obtain formulations with better dissolution profiles in water and, at the same time, with the same safety of the initial drug. Such a strategy can be potentially generalized to other drug–metabolite and prodrug–drug systems with low solubility.

## 2. Materials and Methods

Samples of medicinal-grade nordazepam were kindly provided by Bouchara-Recordati (Puteaux, France), while medicinal-grade diazepam was kindly supplied by Neuraxpharm (Sant Joan Despí, Spain). These two benzodiazepine compounds, with purities higher than 99.5%, were used as received, without further purification. Mechanical binary mixtures at different compositions were prepared by placing the crystalline powders together in a mortar and gently crushing the mixture for a few minutes. The concentration of the so-prepared mechanical binary mixtures varied from 0.05 molar fraction of nordazepam (x_N_) to 0.95. The mechanically mixed powder samples were heated above their melting points and subsequently cooled directly inside the experimental equipment for calorimetric or dielectric characterization to the lowest characterization temperatures, as detailed below, to obtain amorphous glassy mixtures.

Differential scanning calorimetry (DSC) experiments were carried out at a heating/cooling rate of 10 K min^−1^ on both crystalline and amorphous samples, by means of a Q100 thermal analyzer from TA Instruments (New Castle, DE, USA), equipped with an intracooler system that allows it to work in a controlled nitrogen atmosphere, with a flow rate of 25 mL/min, and reaching temperatures as low as 183 K. The experimental temperature range for DSC measurements on the studied samples was between 273 and 493 K. Samples had typical masses of ca. 5 mg and were loaded in pierced aluminum pans. Details of the temperature and enthalpy calibration of the device are available in an earlier publication [12]. The transition temperatures were determined either from the low-temperature onset of the calorimetric features (for pure compounds and for the eutectic point) or from the high-temperature onsets (for the liquidus line). In the latter case, the typical width of melting features (onset-to-onset) was subtracted to allow for direct comparison [13]. Enthalpies of transitions were determined from the area under the thermograms.

Thermogravimetry analysis (TGA) scans were performed while heating the sample under N_2_ flow between room temperature (300 K) and 600 K, at a rate of 10 K min^−1^, by means of a Q50 thermobalance from TA-Instruments. Fourier-transform infrared (FTIR) spectra were acquired at room temperature, in the mid-infrared range (4000–400 cm^−1^), by means of a Nicolet 6700 spectrophotometer equipped with a He/Ne laser source and DTGS-CsI detector and controlled by the Omnic software. Each spectrum was the average of 512 scans collected with 1 cm^−1^ in attenuated total reflection (ATR) mode on samples placed on the surface of a Ge crystal. The baseline of the resulting spectra was corrected, and an automatic smoothing was applied.

Complex permittivity and conductivity spectra of the amorphous binary mixtures were recorded by means of broadband dielectric spectroscopy (BDS), using a Novocontrol analyzer (10^−2^ to 10^7^ Hz) equipped with a Quatro temperature controller with an error better than 0.3 K. The powder samples were placed in a stainless-steel parallel-plate capacitor designed especially for liquids, with the electrodes kept at a fixed distance by cylindrical silica spacers of 50 μm in diameter. The crystalline powders were melted directly inside the capacitor. Isothermal dielectric spectra were acquired in a routine where the temperature was increased between spectra in steps of either 2 or 5 K.

We employed the Grafity^®^ software to fit the dielectric spectra as the sum of a power law representing the dc conductivity contribution and several components each representing a dielectric relaxation process. The complex permittivity was thus modeled as follows:

(1) $$\varepsilon^{*}\left(\omega \right)=\frac{\sigma }{{\left(i\omega \right)}^{s}}+\frac{\Delta \varepsilon_{\alpha }}{{\left(1+{\left(i\omega \tau_{HN,\alpha }\right)}^{a_{\alpha }}\right)}^{b_{\alpha }}}+\underset{k}{\sum }\frac{\Delta \varepsilon_{k}}{1+{\left(i\omega \tau_{k}\right)}^{a_{k}}}+\varepsilon_{\infty }$$

where ω = 2πν is the angular frequency, and ε_∞_ is the permittivity in the high-frequency limit. The first term on the right-hand side is the dc conductivity contribution, which was characterized by an exponent *s* close to unity. The second term is a Havriliak–Negami (HN) function [14] representing the so-called structural (α) relaxation of the sample, whose kinetic slowdown marks the glass-transition temperature. In this function, Δε*_α_* is the dielectric intensity (or relaxation strength) of the α relaxation, *a_α_* and *b_α_* are parameters describing the shape of the structural loss curves (imaginary part of the permittivity), and τ_HN,α_ is a time parameter from which the characteristic relaxation time (τ_α_) where the structural relaxation loss is maximum is as follows:

(2) $$\tau_{\alpha }=\tau_{HN,\alpha }{\left[\sin\left(\frac{a_{\alpha }\pi }{2b_{\alpha }+2}\right)\;\;\right]}^{-1/a_{\alpha }}{\left[\;\sin\left(\frac{a_{\alpha }b_{\alpha }\pi }{2b_{\alpha }+2}\right)\;\right]}^{1/a_{\alpha }}$$

The last term in Equation (1) is a sum of several Cole–Cole functions [15], one for each secondary relaxation process, where Δε*_k_* is the dielectric intensity of the *k*-th secondary relaxation, *a_k_* is the corresponding Cole–Cole exponents, and τ*_k_* is the loss maximum. The shape parameters *a_α_*, *b_α_*, and *a_k_* were free to vary between 0 and 1. Most dielectric spectra displayed only two or, at the most, three relaxations in the accessible frequency window.

First-principles calculations based on density functional theory (DFT) [16] were performed based on the PBE functional [17] with the VASP software [18]. Long-ranged dispersion interactions were captured by using three different van der Waals DFT approaches, namely Grimme’s DFT-D3 [19], many-body dispersion energy (MBD) [20], and Tkatchenko-Scheffler with iterative Hirshfeld partitioning (TS–HP) [21]. The following electronic states were considered to be valence: Cl *s*^2^*p*^5^, O *s*^2^*p*^4^, N *s*^2^*p*^3^, C *s*^2^*p*^2^, and H *s*^1^. Periodic boundary conditions were applied along the three Cartesian directions, and to avoid spurious interactions between neighboring images, large-size supercells of 40 Å × 40 Å × 40 Å were employed in the simulations. Wave functions were represented in a plane–wave basis truncated at 650 eV, and a Γ-centered k-point grid of 2 × 2 × 2 was employed for integrations within the Brillouin zone. Geometry relaxations were performed to optimize the positions of the atoms, considering an atomic force threshold of 0.005 eV/Å. We started from several different initial arrangements of the diazepam–nordazepam dimer and relaxed them, thereby identifying five different dimer conformations corresponding to local energy minima. In order to estimate the density of vibrational states at the center of the Brillouin zone (i.e., Γ reciprocal space point), we employed the density functional perturbation theory as it is implemented in the VASP code. The interaction energy of the heteromolecular dimer, *E*_int_, was straightforwardly estimated with the following formula:

*E*_int_ = *E*_dimer_ − *E*_diazepam_ − *E*_nordazepam,_ (3)

where *E*_dimer_ is the total energy of the relaxed dimer, and *E*_diazepam_ and *E*_nordazepam_ are the energies of the corresponding relaxed molecular species (isolated molecule).

## 3. Results and Discussion

### 3.1. Calorimetric Assessment of the Binary Phase Diagram

**Figure 1:**
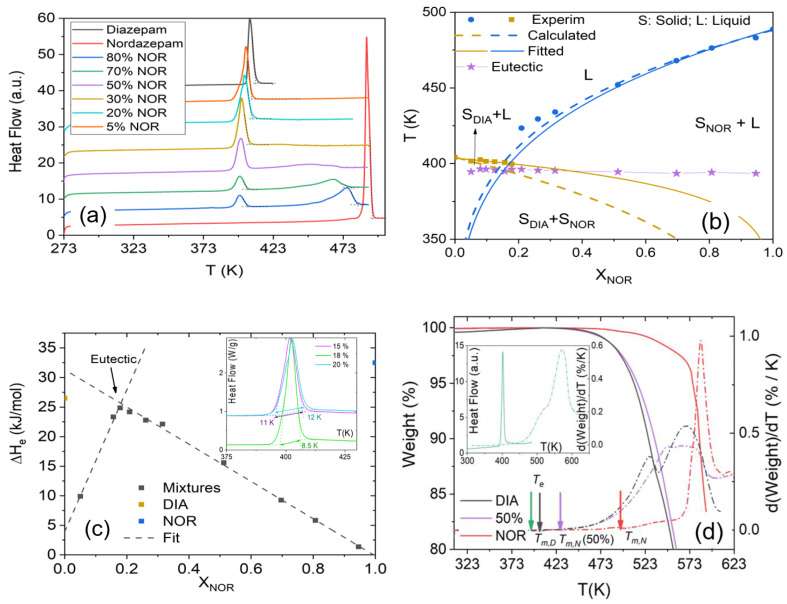
(**a**) First heating DSC traces of DIA, NOR, and their binary mixtures at distinct molar fractions, where the eutectic peak, as well as melting peaks corresponding to the phase separated components, can be observed. (**b**) Equilibrium-phase diagram, as a function of NOR molar fraction, for the binary system composed of DIA and NOR. The panel shows experimental data from DSC measurements (solid markers), the predicted liquidus temperatures with the Schröder–van Laar–Le Chatelier Equation (4) (dashed lines), and guides to eye which are fits of the experimental points with an equation linear in 1/*T* (continuous line). (**c**) Eutectic melting enthalpy as a function of NOR molar fraction (Tammann diagram). The arrow indicates the eutectic concentration. Lines are a guide to the eye. Inset: Comparison of melting peaks for the mixtures with NOR molar fraction x = 0.15, x = 0.18, and x = 0.20. (**d**) TGA curves of pure NOR and DIA, and of the equimolar mixture. Arrows indicate the onset melting point of all three samples. Inset: DSC (continuous line) and TGA (dashed line) traces of the eutectic mechanical mixture.

The result of the mechanical mixing process can be characterized by analyzing the DSC traces, a selection of which is shown in Figure 1a. The full series of thermograms is available in the Supplementary Material file, Figures S1–S13. The bottom and top thermograms shown in Figure 1a correspond, respectively, to pure crystalline nordazepam (hereafter, NOR), which displays a melting point at 487 K, and pure crystalline diazepam (hereafter DIA), which melts at 404 K. In most DSC traces of the mechanically obtained crystalline mixtures, two melting peaks are observed, namely the eutectic peak at lower temperature, corresponding to the melting of the eutectic crystalline mixture (see below), and the so-called liquidus peak at higher temperature, corresponding to the melting of the NOR- or DIA-rich solid phase.

The resulting equilibrium-phase diagram (see Section 2 for technical details) is depicted in Figure 1b, where both melting peaks are displayed as a function of concentration. The shape of the diagram is the typical one for eutectic mixtures of immiscible crystalline components, with the coexistence of solid and liquid phases between the eutectic line (common eutectic temperature, stars) and the liquidus line (final melting point, filled squares) [22]. The first melting peak at lower temperature is, in fact, virtually independent of temperature and corresponds to the eutectic melting point. Averaging the melting onset for the different concentrations studied yields a eutectic point of *T*_e_ = 395.4 ± 1.2 K, which is lower than the melting temperatures of either of the pure solids. The liquidus line, with a final melting point that decreases as one moves away from the pure compounds, indicates the typical melting-point depression that occurs when impurities are added to a pure substance or when, as in this case, the entropic contribution to the free energy of the liquid mixture enlarges its temperature range of stability compared to the pure crystalline solids.

As visible in Figure 1a, the eutectic composition has a single melting peak. In fact, the eutectic composition has the unique property that it remains unchanged also when the eutectic binary mixture is melted or crystallized (in contrast, the compositions of the two phases coexisting between the eutectic and liquidus line, especially the liquid phase, vary with temperature in the same interval).

We found no experimental evidence for a tendency of the two drugs to mix in the crystalline state. The two involved molecules differ by the substitution of a methyl group linked to a nitrogen atom of the diazepinic ring in DIA by a single hydrogen atom in NOR, thus forming a primary amine. As reported in a previous study by some of us, this modification leads to the presence of H-bond interactions in crystalline NOR (while they are fundamentally absent in DIA) and thus to a higher melting point in NOR and in significant differences between the two crystal structures [23], thus rationalizing the lack of miscibility in the crystalline state. On the other hand, it is shown in the following sections that the extent of hydrogen bonding in the liquid phases is comparatively small, and that the main interaction arises from dispersion forces. Therefore, it is possible that the similar chemical structure of DIA and NOR leads to similar dispersion interactions, and that this facilitates the formation of a liquid eutectic mixture, in the sense that there is no large enthalpic contribution to oppose mixing in the liquid state.

The final phase-transition temperatures (liquidus lines) can be theoretically calculated for every mixture by assuming the simplified Schroeder–van Laar–Le Chatelier equation [24], which describes the behavior of binary systems that are immiscible in the crystalline state and show ideal mixing in the liquid state:

(4) $$ln\left(x_{j}\right)=-\frac{\Delta H_{j}{}^{0}}{R}\left(\frac{1}{T_{m,j}}-\frac{1}{T_{m,j}{}^{0}}\right)$$

where *x_j_* is the molar fraction of the *j* component (where either *j* = DIA or *j* = NOR); $\Delta H_{j}{}^{0}$ and $T_{m,j}{}^{0}$ are the melting enthalpy (measured in J mol^−1^) and the melting temperature (in K) of the pure compounds, respectively; and $T_{m,NOR}$ and $T_{m,DIA}$ are the melting temperatures of nordazepam- and diazepam-rich phases of the binary mixture (for completely immiscible solids, these are basically pure crystalline NOR domains and pure crystalline DIA domains, respectively). The dashed lines in Figure 1b correspond to the predictions of the Schroeder–van Laar–Le Chatelier Equation (4). Although the latter do not coincide perfectly with the experimentally measured temperatures, the difference at concentrations close to either of the pure compounds is compatible with the experimental uncertainty in the determination of the melting enthalpies of the pure components in the mixtures. As guides to the eye, we also show fits of the experimental data points with functions of the form *a* + *b*/*T*, i.e., linear in the inverse temperature (continuous lines).

The experimental determination of the eutectic composition is obtained by considering the variation with sample composition of eutectic enthalpy, i.e., the experimental transition enthalpy at *T*_e_ (area under the DSC trace in the region of the lower-temperature transition) as a function of composition. The eutectic enthalpy of the mechanically obtained binary samples as a function of concentration (Tammann diagram) is displayed in Figure 1c. It can be observed that it reaches a maximum for x_NOR_ = 0.18, which coincides with the intercept of two straight-line fits (dashed lines in Figure 1c). As visible in the inset to Figure 1c, where we compare the line shape of the x_NOR_ = 0.18 sample with those of the x_NOR_ = 0.15 and 0.20 mixtures, the eutectic composition displays the smallest width of melting feature, as expected from the coincidence of eutectic and liquidus lines for this sample. The experimental eutectic molar fraction of x_NOR_ = 0.18 implies that the eutectic composition corresponds to a ratio of DIA to NOR molecules between 4:1 and 5:1.

The sample at the eutectic composition obviously exhibits the lower melting point of all binary mixtures. This aspect is important because NOR decomposes upon melting, as is visible in the thermogravimetry scans displayed in Figure 1d, which compares the curves measured on the pure compounds and the equimolar mixture (x_NOR_ = x_DIA_ = 50%). The onset of intense mass loss in pure NOR is above 500 K, but a (weaker) onset of mass loss is detected already at the melting point of this compound. Degradation of pure NOR in the liquid phase is confirmed by the fact that the compound acquires color (see below). On the other hand, the TGA trace of pure DIA displays a constant mass in a relatively large temperature range near the melting point of this compound. This shows that the vapor pressure of DIA is negligible at *T*_m_. The TGA curve of pure DIA and of the equimolar mixture both display an onset of the mass loss at yet a lower temperature (470 K) than NOR. This temperature is well above the melting point of pure DIA, which, in fact, is known to be chemically stable upon melting and beyond and to degrade only at a much higher temperature.

The most important observation is that the eutectic melting temperature (T_e_ = 395.4 ± 1.2 K) lies more than 100 K below the melting point of pure NOR and more than 70 K below the onset of mass loss of either of the pure compounds and of the mixtures. This is visible experimentally in the inset to Figure 1d, where we showed, in the same graph, the DSC trace and the derivative of the TGA trace of the eutectic sample, which confirms visually that the eutectic melting endotherm takes place at significantly lower temperature than any mass loss that might be related to sublimation or decomposition of the sample (in the main panel, the position of the liquidus line is shown for the equimolar mixture in order to allow for a comparison with the TGA derivative). This indicates that the liquid eutectic NOR–DIA mixture can be obtained by simple annealing without any degradation of the two drugs, as further discussed below.

**Figure 2:**
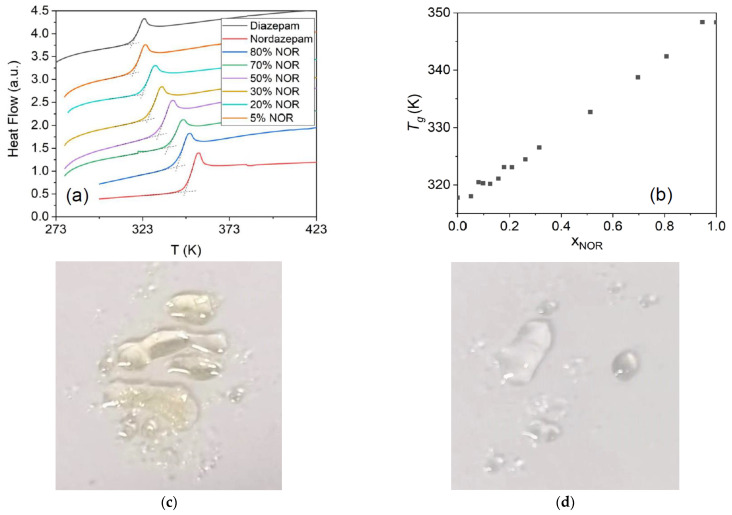
(**a**) Second heating DSC traces of DIA, NOR, and their binary mixtures at different molar fractions: only the glass-transition step is visible. (**b**) Glass-transition temperatures of DIA and NOR binary mixtures as a function of NOR molar fraction. (**c**,**d**) Room-temperature photographs of a droplet of glassy NOR melted just above *T*_m,N_ (**c**), and of an eutectic droplet obtained by melting the x_NOR_ = 0.18 mechanical mixtures to the eutectic melting point *T*_e_ (**d**). The powders were melted directly on a glass slide.

In order to obtain liquid mixtures and supercool them to study the nonequilibrium-phase diagram, all the samples presented in Figure 1 were heated above their corresponding liquidus lines and then rapidly cooled to below room temperature at a rate of 10 K/min to reach the glass state. Selected DSC thermograms acquired upon heating the resulting glassy mixtures are displayed in Figure 2a (the whole series of traces is available in the Supplementary Information file). All curves display a single step-like feature, signaling the discontinuity in specific heat across the glass-transition temperature (*T*_g_), which is unique for a given concentration. In the case of the pure NOR sample, the *T*_g_ obtained with our procedure is indicative of the true glass transition of these samples, despite the possible presence of impurities caused by thermal degradation, since the latter does not alter the *T*_g_ value much [25]. The glass transition was accompanied in all cases by a small enthalpy recovery peak due to the ageing of the glassy mixtures. The onset *T*_g_ temperatures are shown as a function of NOR molar fraction in Figure 2b, where it may be observed that the *T*_g_ varies linearly with composition. The linear dependence of *T*_g_ upon the composition confirms the ideal behavior of the DIA–NOR mixtures.

As discussed above, the melting temperature of the eutectic concentration is lower than any onset of mass loss in TGA and more than 100 K below the melting point of NOR, which suggests that, while pure NOR degrades upon melting, this effect is absent in our mixtures. The effect of thermal degradation of NOR upon melting is visible in Figure 2c, where it can be observed that pure NOR acquires a yellowish color upon melting in air (we observed, instead, a dark blue color for the degradation product when NOR was heated in a sealed heat-resistant glass vessel (not shown)). In contrast, the eutectic mixture is colorless above the eutectic point, as is visible in Figure 2d. This further proves the thermal stability of the amorphous eutectic NOR–DIA mixture and confirms that glassy NOR–DIA samples can be obtained by a simple heating procedure.

No melting or cold-crystallization peak is observed for any of the concentrations studied in subsequent DSC experiments below or above *T*_g_, thus indicating that the amorphous binary samples did not show a strong tendency toward recrystallization. In fact, we obtained amorphous binary mixtures which were kinetically stable against recrystallization for long periods of time (at least weeks) for all molar fractions, which is impressive since even molar concentrations as low as 5% appeared to be sufficient to kinetically stabilize the amorphous binary mixture. In particular, the eutectic glass mixture was found to be kinetically stable for at least 2 months at room temperature. The nature of intermolecular interactions and their possible role for both the miscibility and kinetic stability of the amorphous mixtures are investigated in Section 3.3.

### 3.2. Dielectric Characterization of Amorphous Mixtures

We employed dielectric spectroscopy to characterize the electric properties and molecular dynamics of the pure amorphous compounds, as well as two amorphous binary mixtures with different molar fractions, namely the eutectic concentration (taken to be x_NOR_ = 0.18 based on our enthalpy measurements) and the equimolar mixture (x_NOR_ = 0.50). This second concentration was chosen to better study the effect of heteromolecular (NOR–DIA) interactions.

**Figure 3:**
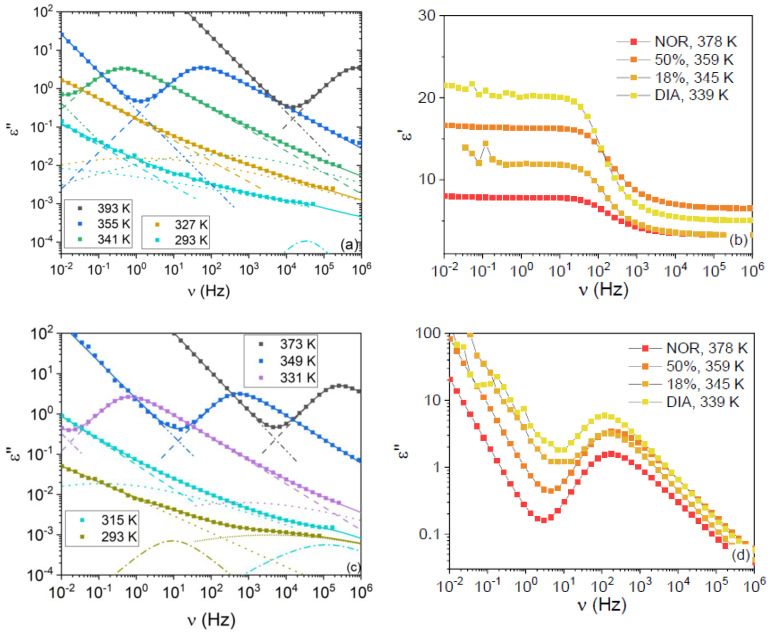
(**a**,**c**) Dielectric loss spectra of the equimolar (**a**) and eutectic (**c**) amorphous mixtures at selected temperatures. Markers are experimental points, continuous lines are fits, and dashed or dotted lines are fit components (each color represents a different temperature). (**b**,**d**) Real and imaginary part, respectively, of the dielectric permittivity of DIA, NOR, and binary mixtures at selected temperatures (see legend), chosen so that the relaxation time of the primary relaxation was the same in all spectra.

Typical imaginary permittivity spectra of the two studied mixtures are displayed in Figure 3a,c for the mixtures with x_NOR_ = 0.18 and x_NOR_ = 0.50, respectively. The most intense relaxation in the spectra acquired above the *T*_g_ of the samples is the primary α relaxation, while secondary relaxations (which are labeled as β, γ, and γ’ in order of increasing relaxation frequencies) can be observed as shoulders to the latter peak or as broad low-intensity features below *T*_g_. The α relaxation followed the expected behavior with sample composition at fixed temperature, being peaked at a frequency intermediate between that of the pure compounds, in agreement with our calorimetric *T*_g_ measurements. In correspondence with the α relaxation peak of the imaginary permittivity, the real permittivity displays a step-like decrease from its static value (ε_s_, the zero-frequency limit of the real part of the dielectric function) to its high-frequency value (where “high frequency” is defined based on the available experimental range in dielectric spectroscopy).

Figure 3b,d display the comparison between the real and imaginary permittivity spectra of the four studied samples, at different temperatures chosen, so that the α relaxation was peaked at roughly the same frequency in all spectra. It can be observed in Figure 3d that the dielectric strength of the α relaxation is similar for both mixtures and is, in both cases, intermediate between those of the pure DIA and NOR liquids. Nevertheless, the static permittivity is different, as is visible in Figure 3b, with the ε_s_ of the x_NOR_ = 0.18 sample being lower than that of the x_NOR_ = 0.50 sample and closer to the static permittivity of pure liquid NOR. As visible in the plot, such a higher value of the static dielectric permittivity is actually associated with a higher value of the high-frequency dielectric function, rather than with an increase of the orientational polarization contribution. The origin of such an increase in the high-frequency response is beyond the scope of this contribution.

**Figure 4:**
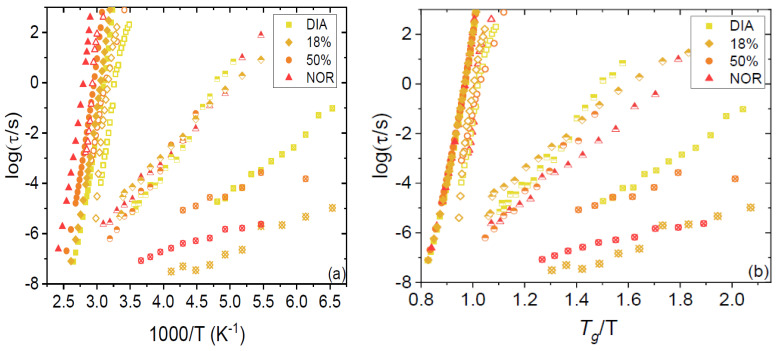
Relaxation times of DIA, NOR, and the 18% and 50 % binary mixtures (see legend) plotted against the reciprocal of the temperature (Arrhenius plot, (**a**)) and of the reduced temperature *T*/*T*_g_ (Angell plot, (**b**)). All relaxations are shown: α (solid points), β (open points), γ (half points), and γ’ (crossed points).

All spectra were fitted by using the procedure detailed in Section 2. The obtained relaxation times for both the primary and secondary relaxations are plotted as Arrhenius and Angell relaxation maps in Figure 4a,b, respectively. It may be observed that both the α (filled markers) and β relaxations (open markers) are well superposed in the Angell representation. The first finding indicates that the so-called kinetic fragility (which is a measure of the curvature of the Angell plot at *T*/*T*_g_ = 1) of both amorphous compounds, as well as their binary mixtures, is very similar (see below). The superposition of β relaxation times indicates that such a relaxation process is the subdiffusive Johari–Goldstein relaxation, which represents the local single-molecule analogue of the cooperative α relaxation [26,27,28].

By convention, the temperature at which the α relaxation time reaches a characteristic time of 100 s is defined as the dynamic *T*_g_. The dynamic *T*_g_ values for the 18% and 50% amorphous mixtures were 317 and 328 K, respectively, values that are very close to the calorimetric glass-transition temperatures (see Table 1) of the same samples. The temperature dependence of the α relaxation could be well described by the Vogel–Fulcher–Tamman function [29,30,31], which is typical of cooperative structural relaxations [32]:

(5) $$\tau_{\alpha }\left(T\right)=\tau_{0}\exp\;\left(\frac{DT_{VF}}{T-T_{VF}}\right)$$

The fit parameters appearing in Equation (5) are reported in Table 1 for the four samples investigated. From these parameters, the so-called kinetic fragility, *m_p_* [33,34], is determined as follows:

(6) $$m_{p}={\left[\frac{d}{d\left(T_{g}/T\right)}\log\left(\tau_{\alpha }\right)\;\right]}_{T=T_{g}}=\frac{DT_{VF}T_{g}}{{\left(T_{g}-T_{VF}\right)}^{2}\;ln10}$$

As is visible in the table, the fragility of all studied samples was virtually the same and roughly equal to 71 (average value). This value is in agreement with previously found values for the pure compounds [23,35], and its constancy is in line with the ideal behavior of the DIA–NOR liquid mixture (see [36] for an example of non-ideal behavior of the fragility index).

The secondary processes displayed, instead, a simply activated (Arrhenius) behavior. The corresponding activation energies are reported in Table 1. The order of magnitude of the activation energies for the mixtures is in line with previous dielectric spectroscopy studies on the pure compounds. They are all a factor of two larger than the activation energy reported in a recent study of the pure compounds by means of thermally stimulated currents’ characterization [35].

Only one Johari–Goldstein relaxation was detected, as may be expected for a homogeneous liquid mixture. In fact, the other relaxations visible in Figure 4 were previously observed in the pure compounds, where they were assigned to intramolecular relaxation modes. In particular, the γ process (half-filled markers) is present in both pure DIA and NOR, where it was unambiguously identified as the inter-conformational relaxation process corresponding to the ring inversion dynamics of the seven-membered diazepinic ring [23]. As visible in the Arrhenius plot of Figure 4a, such inter-conformer relaxation dynamics takes place at basically the same rate in all four studied samples; that is, its characteristic time is basically independent of composition for a fixed temperature.

Moreover, the fastest process (crossed markers) was detected previously in the pure compounds [23]. The fastest relaxation of DIA is shown here to be visible also in the eutectic DIA–NOR mixture, while the fastest relaxation of NOR is detected also in the equimolar mixture. These fast relaxations were tentatively assigned to rigid small-angle rotations of the (fused-ring) benzodiazepinic structure about its bond with the phenyl ring. This interpretation may hold in the case of pure DIA, where this process is significantly slower and displays a higher activation energy compared to NOR (Figure 4a). In the latter compound, instead, the very short relaxation time, the low activation energy, and the low spectral intensity of this feature all suggest that the origin of this relaxation might be associated with the rotation of the benzene ring itself. In fact, although such a conjugated ring possesses per se no dipole moment, a change in its orientation might affect the electron distribution in the fused benzodiazepinic ring, which could lead to a small change in the molecular dipole moment and/or hydrogen bonding motif upon rotation of the benzene ring, which would lead to a small but detectable signal in the BDS spectra.

**Figure 5:**
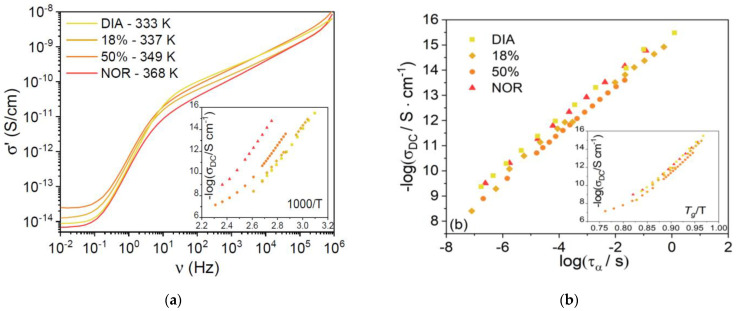
(**a**) Real part of the complex conductivity function against the frequency, at a selected temperature for pure liquid NOR and DIA and for the eutectic and equimolar liquid mixtures. Temperatures are indicated in the legend. Inset: Arrhenius plot of the temperature dependence of the DC conductivity (σ_DC_) of the same samples. (**b**) Logarithm of σ_DC_ of the same samples against their structural (α) relaxation time. Inset: Angell plot of σ_DC_.

Figure 5a shows typical high-temperature AC conductivity spectra (vs. frequency) for each supercooled liquid sample, at temperatures chosen such that the ratio *T*/*T*_g_ was roughly constant and equal to 1.75 ÷ 1.78 for all four samples. The AC spectra display a plateau at low frequency, corresponding to the DC electric conductivity, σ_DC_, of the liquid phases. It can be seen that the σ_DC_ value is very similar for all samples at this rescaled temperature. In Figure 5b, we plot the logarithm of σ_DC_ against the logarithm of the structural relaxation time, τ_α_. A linear correlation is observed in all three compounds, with slopes of 0.93 ± 0.06 for DIA and 0.93 ± 0.02 for NOR. Such values are virtually identical for the two pharmaceuticals studied and very close to the value of 1 expected from the Stokes–Einstein relation [37,38]. This result indicates that the diffusion of charged species is coupled to the α relaxation, rather than to the intramolecular γ and γ’ conformational dynamics, implying that the DC electric conductivity of the amorphous drugs is due to the viscosity-limited diffusion of ionic impurities [39,40,41,42]. The ionic character of the charge conduction is also confirmed by the curvature of the Angell plot of σ_DC_ shown in the inset to Figure 5b. The fact that the DC conductivity is basically the same for both compounds under isochronal conditions. (Figure 5b shows that the microscopic mechanism for ion diffusion is similar in both liquid benzodiazepine derivatives.)

### 3.3. FTIR and DFT Study

**Figure 6:**
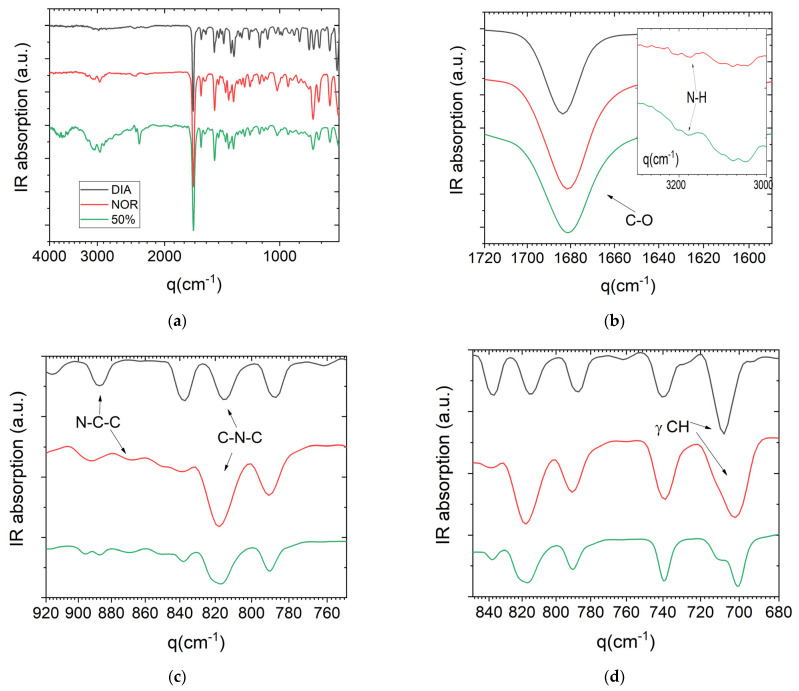
(**a**) Global FTIR spectra of the poly-crystalline DIA and NOR powders and their 50% binary mixture in the glass (amorphous solid) state. (**b**–**d**) Zoom of the spectra of panel (**a**) in the spectral region of the C=O vibration (carbonyl group, b), C–N modes, and C–H vibrations of the chlorobenzene ring, respectively. Inset to (**b**): detail of the spectra in the region of the (hydrogen-bonded) N–H stretching.

We employed FTIR spectroscopy and DFT simulations to obtain information on the interactions between the two pure APIs in the amorphous mixture. Figure 6 shows the room-temperature FTIR spectra of the pure DIA and NOR powders and of the 50% glassy binary mixture acquired in reflection geometry (ATR). Overall, the spectrum of the 50% mixture appears rather similar to both pure components, and in particular to that of NOR. This is confirmed by the frequencies of the assigned bands, when compared to the bands of the pure APIs (we followed the assignment provided by Neville et al. [43]).

Table 2 lists some selected bands and their corresponding assignments. In the amorphous 50% mixture, the most intense feature of the spectrum is the carbonyl stretch at 1680 cm^−1^. This wavenumber is very similar to that of the same vibrational mode in NOR and slightly lower than that of DIA (1683 cm^−1^). Neville et al. [43] suggested that the change in frequency between NOR and DIA, and the larger width of the peak in NOR, are both due to the hydrogen bonding between the carbonyl and the amide group of NOR in the crystalline compound (hydrogen bonds are obviously absent in crystalline DIA). The presence of such H-bonds in crystalline NOR was confirmed experimentally [23]. It is possible that a similar interaction is present also in a fraction of the molecules in the equimolar mixture, where it arguably also involves the carbonyl group of DIA molecules. The C=O band is rather broad in all samples, and this does not allow us to rule out the presence of distinct spectral components (for example, in the binary mixture at the frequency of the non-hydrogen-bonded C=O stretch of pure DIA).

The amidic nitrogen of NOR is the only H-bond donor group in either molecule, and both of the dilutions of NOR species in the amorphous binary mixtures and their intrinsic disordered nature indicate that H-bonding interactions are necessarily weaker in the amorphous mixtures than in pure crystalline NOR. Pure NOR displays a weak band corresponding to the stretching of the N–H bond at 3174 cm^−1^ (inset to Figure 5b), which is typical of amides. The same band is also observed, but with a higher intensity and slightly shifted, in the glassy 50% mixture (3176 cm^−1^), while it is absent (as expected) in DIA. It is possible that the shift of the N–H vibration in the liquid binary sample is due to a lower impact of hydrogen bonding, as may be expected from the intrinsic disorder of an amorphous phase. Correspondingly, non-directional (e.g., van der Waals) interactions are expected to play an important role in the liquid mixture.

The N–C–C stretching bands of the pristine compounds (observed at 887 cm^−1^ in DIA and 867 cm^−1^ in NOR, respectively), appear at the same frequency in the binary mixture. The band attributed to the CH_3_ twisting in DIA is also preserved, even though it is found to be less intense, likely due to the lower amount of DIA. On the other hand, the 50% mixture displays two bands at 815 and 821 cm^−1^ in the region of the C–N–C stretching, which are observed, respectively, at 815 cm^−1^ in pure DIA and 818 cm^−1^ in NOR. The change in C–N–C stretching between pure NOR and the 50% mixture suggests that intermolecular interactions in the equimolar DIA–NOR mixture may involve also the diazepinic skeletal nitrogen (which is characterized by an excess local negative charge due to the presence of a lone electron pair, which may be involved in hydrogen bonding but also in ring–ring interactions involving diazepinic moieties and/or the electron-rich π orbital of the conjugated benzene rings). In the spectra of the 50% mixture, the two strong IR bands near 700 cm^−1^ and the weaker band at 896 cm^−1^ were observed to be significantly shifted with respect to the corresponding features in the two pure crystalline compounds. These bands are assigned to out-of-plane CH bending modes characteristic of monosubstituted benzene derivatives, and their shift with respect to the pure compounds provides support for the occurrence of interactions involving π benzene electrons in the binary amorphous mixture.

**Figure 7:**
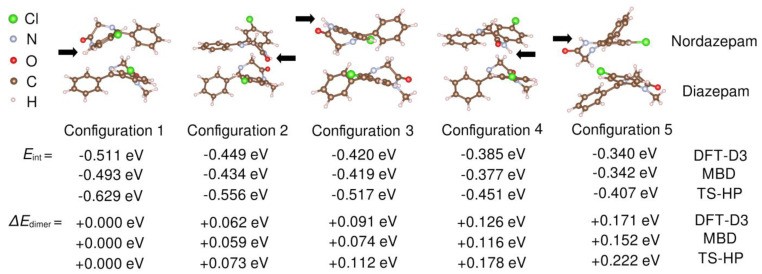
DFT interaction energies, *E*_int_, estimated for several heteromolecular dimer configurations. Results were obtained with three different van der Waals DFT functionals, namely DFT-D3, MBD, and TS-HP. The dimer energies are referred to as “Configuration 1”, the most stable molecular arrangement systematically found in our vdW-DFT simulations, Δ*E*_dimer_ = *E*_Configuration x_ − *E*_Configuration 1_. The positions of the Nordazepam amidic nitrogen are indicated with black arrows.

First-principles simulations based on van der Waals–corrected density functional theory (vdW-DFT) calculations were performed to theoretically characterize the interactions and vibrational modes of the heteromolecular dimer (see Section 2 for technical details). Since the FTIR results, as well as the chemical structure of the molecules, suggest the possible existence of important dispersion interactions between ring moieties, we started from arrangements of a NOR–DIA dimer with a rough coplanarity of at least some rings. After allowing these arrangements to relax into local energy minima, we identified five distinct molecular dimer arrangements of minimal energy (Figure 7), in which the nordazepam amidic nitrogen (N–H) either faced (Configurations 1, 2, and 4) or opposed (Configurations 3 and 5) the diazepam framework. In all the analyzed cases, the calculated (relaxed) heteromolecular dimer interaction energy, *E*_int_ (see Equation (3) in Section 2), was of negative sign and equal to several tenths of an eV in absolute value. For instance, with the TS–HP van der Waals approach, we estimated an *E*_int_ of −0.629 and −0.407 eV for Configurations 1 and 5, respectively. The origin of such large and negative interaction energies in our vdW-DFT simulations is purely electrostatic (see below). Our computational results clearly indicate the presence of relatively strong attractive forces among the ring moieties of NOR and DIA molecules. The five heteromolecular dimer configurations shown in Figure 7 are ordered according to their zero-temperature energy: “Configuration 1” was systematically found to be the most stable, and “Configuration 5” the least stable, with the dimer energy of the latter being about 0.2 eV larger than that of the former. In the two energetically most favorable dimer configurations, the amidic nitrogen of NOR faces the DIA molecule (“Configuration 1 and 2”), thus giving plausibility to the formation H bonds between the two molecules; on the other hand, in the energetically less favorable dimer configuration (“Configuration 5”), the amidic nitrogen opposes the DIA moiety, hence precluding the formation of H bonds among the two molecules. Our simulations do not provide explicit evidence for H bonding in the heteromolecular dimer; in particular, in the relaxed geometries, the Nordazepam amidic nitrogen always was located at distances larger than 3–4 Å from the diazepam carbonyl oxygen and skeletal nitrogen. This might be due to the well-known limitations of DFT methods in accurately describing H-bonding interactions in small molecules [44,45], but it could also indicate that directional H-bonds are not the dominant interactions between the benzodiazepine moieties in the liquid phase. In either case, the formation of (dynamic) heteromolecular dimers as those reported by our DFT study confirms the chemical affinity of the two benzodiazepine derivatives, which is likely partially responsible for the observed physical stability of the amorphous DIA–NOR mixtures.

**Figure 8:**
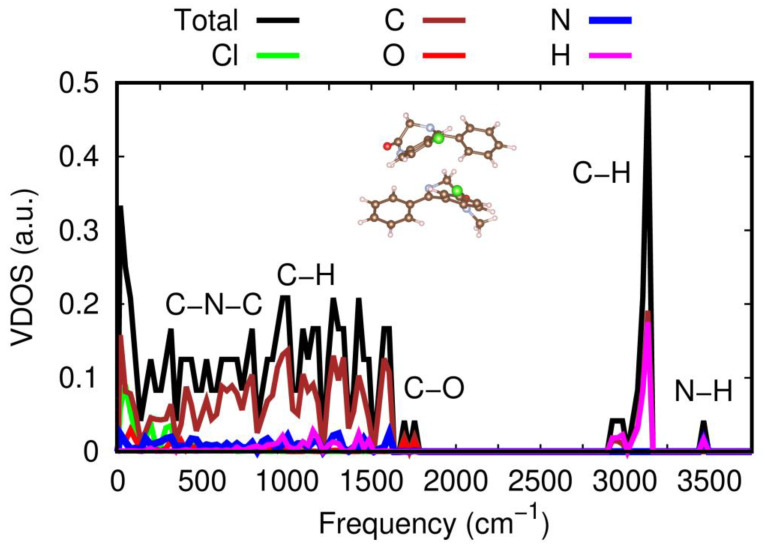
Vibrational density of states, VDOS, calculated with vdW-DFT methods for the energetically most favorable heteromolecular dimer (“Configuration 1”, sketched in the figure). Total and atomic partial VDOS are represented in the figure. Different regions of the vibrational spectrum are highlighted in terms of the atomic species that dominate the corresponding dimer vibrations (that is, they present the largest partial VDOS).

The full vibrational density of states, VDOS, of the energetically most favorable heteromolecular dimer was also computed with theoretical vdW-DFT methods, as shown in Figure 8. We were able to determine the relative contribution of each atomic species to the molecular vibrations by inspecting the corresponding eigenmodes and making use of their orthonormal relations [46]. The same calculations were repeated for the heteromolecular dimer in the “Configuration 2” arrangement, obtaining identical results (not shown here); therefore, the theoretical conclusions explained in what follows do not appear to be critically dependent on the relative molecular orientation of NOR and DIA molecules. As depicted in Figure 8, five clearly differentiable regions appear in the estimated dimer vibrational spectrum at frequencies higher than 500 cm^−1^ (below this threshold frequency, the vibrational modes are mostly governed by Cl and C atoms). The first region encompasses the frequency range 500 cm^−1^ ≤ ω ≤ 800 cm^−1^, in which a large concentration of molecular vibrations dominated by C and N atoms and, thus, related to C–N–C stretching modes are observed. Next, in the frequency interval 800 cm^−1^ ≤ ω ≤ 1500 cm^−1^, the total VDOS is moderately large and uniform with the hydrogen atoms taking on a leading vibrational role along with the carbon atoms. Then three narrow VDOS domains emerge around the frequencies 1700, 3100, and 3500 cm^−1^, which are dominated by O–H, C–H, and N–H atoms, respectively. The first of these peaks can be identified with carbonyl C=O stretching modes within each of the two molecules conforming the dimer, while the latter can be identified with intramolecular N–H stretching modes of NOR species. As regards the agreement with the experimental measurements, while the vdW-DFT calculations fail to reproduce N–H stretching frequency experimentally determined at 3175 cm^−1^, possibly due to the abovementioned limitations to reproduce features directly related to H-bonding interactions, intramolecular vibrations such as the carbonyl stretching mode around 1700 cm^−1^ are well reproduced.

## 4. Conclusions

We employed scanning calorimetry and dielectric spectroscopy to establish the equilibrium- and nonequilibrium-phase diagram of the diazepam–nordazepam system. The equilibrium-phase diagram is characterized by eutectic composition corresponding to a molar fraction of nordazepam of 0.18, with eutectic melting point at 395 K. The two compounds are basically immiscible in the crystalline state, as also confirmed by the comparison with the predictions of the Schröder–van Laar–Le Chatelier equation. Concerning the nonequilibrium diagram, the glass-transition temperature of the amorphous mixtures is found to scale linearly with composition. The kinetic stability of the amorphous eutectic mixture was assessed, and it was shown that the amorphous binary diazepam–nordazepam mixture has better chemical and physical stability than pure amorphous nordazepam.

All amorphous mixtures display a structural relaxation time and a Johari–Goldstein relaxation time that are intermediate between those of the pure compounds and scale with composition. They also display a concentration-independent conformational relaxation process corresponding to the ring inversion of the aliphatic diazepinic ring. The DC electrical conductivity of the amorphous samples is ionic and scales with the structural relaxation time (cooperative mobility) and glass-transition temperature, as typically found in glass-forming electrolytes, and obeyed a fractional Walden rule, with a slope slightly lower than one.

The FTIR-spectroscopy characterization of the binary equimolar amorphous mixture indicates the presence of H-bonding, as well as of interactions involving skeletal carbon and nitrogen atoms, which suggest an important contribution of dispersion interactions between ring moieties. Our DFT calculations provide large formation energies of the nordazepam–diazepam dimer, of the order of several tenths of eV, and confirm that ring-ring interactions are the dominant intermolecular forces. It is likely that such coupling leads to the observed miscibility in the liquid state and contributes to enhance the kinetic stability of the binary glassy mixtures against crystallization.

The achievement of kinetically stable amorphous mixtures of an API with one of its metabolites in the human body is an interesting strategy to obtain formulations with better dissolution profiles and, at the same time, the same safety of the initial drug. We propose that such a strategy may be generalized to other drugs and prodrugs with very low bioavailability, for which the kinetic stabilization of amorphous mixtures of drug + metabolite, or even drug + prodrug, may allow for the oral intake of active pharmaceutical ingredients that would not be otherwise commercialized or that would otherwise require a more expensive delivery strategy.

## Figures and Tables

**Table 1:** Glass transition and fit parameters of selected samples.

NOR Molar Fraction	T_g_ and Fragility	VFT Parameters	Ea [kJ mol^−1^]
0	DSC:317.8 ± 0.4 K	Log($\tau_{0}$/[s]) = −21.8 ± 0.4	β:(1.9 ± 0.1)·10^2^
	BDS:312.6 ± 0.2 K *m_p_* = 73 ± 7	*D* = 24.3 ± 1.4 *T*_0_ = 214 ± 3 K	γ: 72 ± 9 γ’: 37 ± 7
0.18	DSC: 323.1 ± 0.4 K	Log($\tau_{0}$/[s]) = −22.0 ± 0.6	β: (1.9 ± 0.2)·10^2^
	BDS: 317.4 ± 0.3 K *m_p_* = 70 ± 9	*D* =27.8 ± 2.2 *T*_0_ = 211 ± 3 K	γ: 58 ± 4 γ’: 24 ± 3
0.50	DSC: 332.7 ± 0.7 K	Log($\tau_{0}$/[s]) = −21.7 ± 0.4	β: (2.0 ± 0.1)·10^2^
	BDS: 328.0 ± 0.2 K *m_p_* = 69 ± 7	*D* = 28.2 ± 1.6 *T*_0_ = 216 ± 2 K	γ: 73 ± 3 γ’: 24 ± 4
1	DSC: 346.8 ± 1.2 K	Log($\tau_{0}$/[s]) = −21.0 ± 1.0	β: (1.8 ± 0.1)·10^2^
	BDS: 347.2 ± 0.2 K *m_p_* = 73 ± 9	*D* = 23.6 ± 1.9 *T*_0_ = 239 ± 4 K	γ: 58 ± 5 γ’: -

**Table 2:** Wavenumbers (cm^−1^) of selected bands in the IR spectra of DIA, NOR, and their equimolar amorphous binary mixture. The assignment of bands is based on Ref. [43].

Description	DIA (cm^−1^)	NOR (cm^−1^)	50% (cm^−1^)
CH (γ, monosubst)	707 s	702 s	701 s 710 m
CH (γ, monosubst)	740 s	739 s	740 s
C–N–C str.	815 s	818 vs	815 s 821 sh
N–C–C str.	887 m	867 m	867 w 887 w
CH (γ)	-	893 s	896 m
CH_3_ twist.	985 m	-	985 m
C=C str.	1604 vs	1606 vs	1606 vs
C=O str.	1683 vvs	1681 vvs	1680 vvs
N–H str.	-	3174 w	3176 m

v = very, s =strong, m = medium, w = weak, sh =shoulder.

## Data Availability

Data will be made available upon request.

## Supplementary Materials

A supporting Information file can be downloaded at https://www.mdpi.com/article/10.3390/pharmaceutics15010196/s1, containing the full set of DSC traces (heat–cool–heat cycle) measured for each studied concentration of the binary diazepam–nordazepam system. Figure S1.: DSC traces of pure diazepam (DIA), Figure S2: DSC traces of DIA with 5% nordazepam (NOR), Figure S3: DSC traces of DIA with 8% NOR, Figure S4: DSC traces of DIA with 10% NOR, Figure S5. DSC traces of DIA with 12.5% NOR, Figure S6. DSC traces of DIA with 15% NOR, Figure S7. DSC traces of DIA with 18% NOR, Figure S8. DSC traces of DIA with 20% NOR, Figure S9. DSC traces of DIA with 30% NOR, Figure S10. DSC traces of DIA with 50% NOR, Figure S11. DSC traces of DIA with 70% NOR, Figure S12. DSC traces of DIA with 80% NOR, and Figure S13. DSC traces of NOR.

## References

[1] Schick C., Wurm A., Mohammed A., Reiter G., Sommer J.U. (2003). Vitrification and Devitrification of the Rigid Amorphous Fraction in Semicrystalline Polymers Revealed from Frequency Dependent Heat Capacity. Polymer Crystallization: Observations, Concepts and Interpretations.

[2] Valenti S., Diaz A., Romanini M., del Valle L.J., Puiggalí J., Tamarit J.L., Macovez R. (2019). Amorphous binary dispersions of chloramphenicol in enantiomeric pure and racemic poly-lactic acid: Morphology, molecular relaxations, and controlled drug release. Int. J. Pharm..

[3] Gupta B. (2002). Heat Setting. J. Appl. Polym. Sci..

[4] Valenti S., del Valle L.J., Romanini M., Mitjana M., Puiggalí J., Tamarit J.L., Macovez R. (2022). Drug-Biopolymer Dispersions: Morphology- and Temperature-Dependent (Anti)Plasticizer Effect of the Drug and Component-Specific Johari–Goldstein Relaxations. Int. J. Mol. Sci..

[5] Kissi E.O., Khorami K., Rades T. (2019). Determination of Stable Co-Amorphous Drug–Drug Ratios from the Eutectic Behavior of Crystalline Physical Mixtures. Pharmaceutics.

[6] Ueda H., Hirakawa Y., Miyano T., Imono M., Yee Tse J., Uchiyama H., Tozuka Y., Kadota K. (2022). Design of a Stable Coamorphous System Using Lactose as an Antiplasticizing Agent for Diphenhydramine Hydrochloride with a Low Glass Transition Temperature. Mol. Pharm..

[7] Loftsson T., Hreinsdoittir D. (2006). Determination of Aqueous Solubility by Heating and Equilibration: A Technical Note. AAPS Pharm. Sci. Tech..

[8] Shayanfar A., Acree W.E., Jouyban A. (2009). Solubility of Lamotrigine, Diazepam, Clonazepam, and Phenobarbital in Propylene Glycol + Water Mixtures at 298.15 K. J. Chem. Eng. Data.

[9] Hadžiabdić J., Elezović A., Hadžović S., Vehabović M. (2013). The solubility—Intrinsic dissolution rate of diazepam and inclusion complexes diazepam with 2-hydroxypropyl-β-cyclodextrin. Int. J. Sci. Technol. Soc..

[10] Mandrioli R., Mercolini L., Raggi M.A. (2008). Benzodiazepine metabolism: An analitycal perspective. Curr. Drug Metab..

[11] Riss J., Cloyd J., Gates J., Collins S. (2008). Benzodiazepines in epilepsy: Pharmacology and pharmacokinetics. Acta Neurol. Scand..

[12] Barrio M., Espeau P., Tamarit J.L., Perrin M., Veglio N., Céolin R. (2009). Polymorphism of progesterone: Relative stabilities of the orthorhombic phases I and II inferred from topological and experimental pressure-temperature phase diagrams. J. Pharm. Sci..

[13] Courchinoux R., Chanh N.B., Haget Y., Tauler E., Cuevas-Diarte M.A. (1988). Use of the “shape factors” as an empirical method to determine the actual characteristic temperatures of binary phase diagrams by differential scanning calorimetry. Thermochim. Acta.

[14] Havriliak S., Negami S. (1967). A complex plane representation of dielectric and mechanical relaxation processes in some polymers. Polymer.

[15] Cole K.S., Cole R.H. (1942). Dispersion and Absorption in Dielectrics II. Direct Current Characteristics. J. Chem. Phys..

[16] Cazorla C., Boronat J. (2017). Simulation and understanding of atomic and molecular quantum crystals. Rev. Mod. Phys..

[17] Perdew J., Ruzsinszky A., Csonka G., Vydrov O., Scuseria G., Constantin L., Zhou X., Burke K. (2008). Restoring the Density-Gradient Expansion for Exchange in Solids and Surfaces. Phys. Rev. Lett..

[18] Kresse G., Furthmuller J. (1996). Efficient iterative schemes for ab initio total-energy calculations using a plane-wave basis set. Phys. Rev. B.

[19] Grimme S., Antony J., Ehrlich S., Krieg H. (2010). A consistent and accurate ab initio parametrization of density functional dispersion correction (DFT-D) for the 94 elements H-Pu. J. Chem. Phys..

[20] Gould T., Lebègue S., Ángyán J.G., Bučko T. (2016). A Fractionally Ionic Approach to Polarizability and van der Waals Many-Body Dispersion Calculations. J. Chem. Theory Comput..

[21] Bučko T., Lebègue S., Hafner J., Ángyán J.G. (2013). Improved Density Dependent Correction for the Description of London Dispersion Forces. J. Chem. Theory Comput..

[22] Oonk H.A.J., Calvet M.T. (2008). Equilibrium Between Phases of Matter. Phenomenology and Thermodynamics.

[23] Valenti S., Barrio M., Negrier P., Romanini M., Macovez R., Tamarit J.L. (2021). Comparative Physical Study of Three Pharmaceutically Active Benzodiazepine Derivatives: Crystalline versus Amorphous State and Crystallization Tendency. Mol. Pharm..

[24] Schröder I. (1893). Über die Abhängigkeit der Löslichkeit eines festen Körpers von seiner Schmelztemperatur. Z. Phys. Chem..

[25] Valenti S., Romanini M., Franco L., Puiggalí J., Tamarit J.L., Macovez R. (2018). Tuning the Kinetic Stability of the Amorphous Phase of the Chloramphenicol Antibiotic. Mol. Pharm..

[26] Ngai K.L., Paluch M. (2004). Classification of secondary relaxation in glass-formers based on dynamic properties. J. Chem. Phys..

[27] Romanini M., Barrio M., Macovez R., Ruiz-Martin M.D., Capaccioli S., Tamarit J.L. (2017). Thermodynamic Scaling of the Dynamics of a Strongly Hydrogen-Bonded Glass-Former. Sci. Rep..

[28] Caporaletti F., Capaccioli S., Valenti S., Mikolasek M., Chumakov A.I., Monaco G. (2019). A microscopic look at the Johari-Goldstein relaxation in a hydrogen-bonded glass-former. Sci. Rep..

[29] Fulcher G.S. (1925). Analysis of recent measurements of the viscosity of glasses. J. Am. Ceram. Soc..

[30] Tammann G., Hesse W. (1926). Die Abhängigkeit der Viscosität von der Temperatur bie unterkühlten Flüssigkeiten. Z. Anorg. Allg. Chem..

[31] Vogel H. (1921). Das temperaturabhängigkeitsgesetz der viskosität von flüssigkeiten. Phys. Z..

[32] Angell C.A. (1988). Structural instability and relaxation in liquid and glassy phases near the fragile liquid limit. J. Non-Cryst. Solids.

[33] Angell C.A. (1985). Spectroscopy Simulation and Scattering, and the Medium Range Order Problem in Glass. J. Non-Cryst. Solids.

[34] Böhmer R., Ngai K.L., Angell C.A., Plazek D.J. (1993). Nonexponential relaxations in strong and fragile glass formers. J. Chem. Phys..

[35] Diogo H.P., Moura Ramos J.J. (2022). TSDC and DSC Investigation on the Molecular Mobility in the Amorphous Solid State and in the Glass Transformation Region of Two Benzodiazepine Derivatives: Diazepam and Nordazepam. J. Pharm. Sci..

[36] Pardo L.C., Barrio M., Tamarit J.L., López D.O., Salud J., Negrier P., Mondieig D. (1999). Miscibility study in stable and metastable orientational disordered phases in a two-component system (CH_3_)CCl_3_+CCl_4_. Chem. Phys. Lett..

[37] Zachariah M., Romanini M., Tripathi P., Tamarit J.L., Macovez R. (2015). Molecular diffusion and dc conductivity perfectly correlated with molecular rotational dynamics in a plastic crystalline electrolyte. Phys. Chem. Chem. Phys..

[38] Zachariah M., Romanini M., Tripathi P., Barrio M., Tamarit J.L., Macovez R. (2015). Self-Diffusion, Phase Behavior, and Li+ Ion Conduction in Succinonitrile-Based Plastic Cocrystals. J. Phys. Chem. C.

[39] Xu W., Angell C.A. (2003). Solvent-free electrolytes with aqueous solution-like conductivities. Science.

[40] Leys J., Wübbenhorst M., Preethy Menon C., Rajesh R., Thoen J., Glorieux C., Nockemann P., Thijs B., Binnemans K., Longuemart S. (2008). Temperature dependence of the electrical conductivity of imidazolium ionic liquids. J. Chem. Phys..

[41] Köhler M., Lunkenheimer P., Loidl A. (2008). Dielectric and conductivity relaxation in mixtures of glycerol with LiCl. Eur. Phys. J. E.

[42] Sippel P., Lunkenheimer P., Krohns S., Thoms E., Loidl A. (2015). Importance of liquid fragility for energy applications of ionic liquids. Sci. Rep..

[43] Neville G.A., Shurvell H.F. (1990). Fourier transform Raman and infrared vibrational study of diazepam and four closely related 1,4-benzodiazepines. J. Raman Spectrosc..

[44] Kannemann F.O., Becke A.D. (2010). van der Waals Interactions in Density-Functional Theory: Intermolecular Complexes. J. Chem. Theory Comput..

[45] Boese A.D. (2015). Density Functional Theory and Hydrogen Bonds: Are We There Yet?. ChemPhysChem.

[46] Cazorla C., Íñiguez J. (2013). Insights into the phase diagram of bismuth ferrite from quasiharmonic free-energy calculations. Phys. Rev. B.

