# Supplementary material for: Eutectic Mixture Formation and Relaxation Dynamics of Coamorphous Mixtures of Two Benzodiazepine Drugs
Source: Pharmaceutics. 2023 Jan 5;15(1):196. doi: 10.3390/pharmaceutics15010196 (PMC9861849; doi:10.3390/pharmaceutics15010196)
Supplement: Supplementary file 1 [file pharmaceutics-15-00196-s001.zip › pharmaceutics-2106848-supplementary.pdf]

Supplementary Materials: Eutectic Mixture Formation and Relaxation Dynamics of Coamorphous Mixtures of Two Benzodiazepine Drugs

Sofia Valenti, Claudio Cazorla, Michela Romanini, Josep Lluís Tamarit, and Roberto Macovez

This Supplementary material contains the full DSC traces of the measured samples.

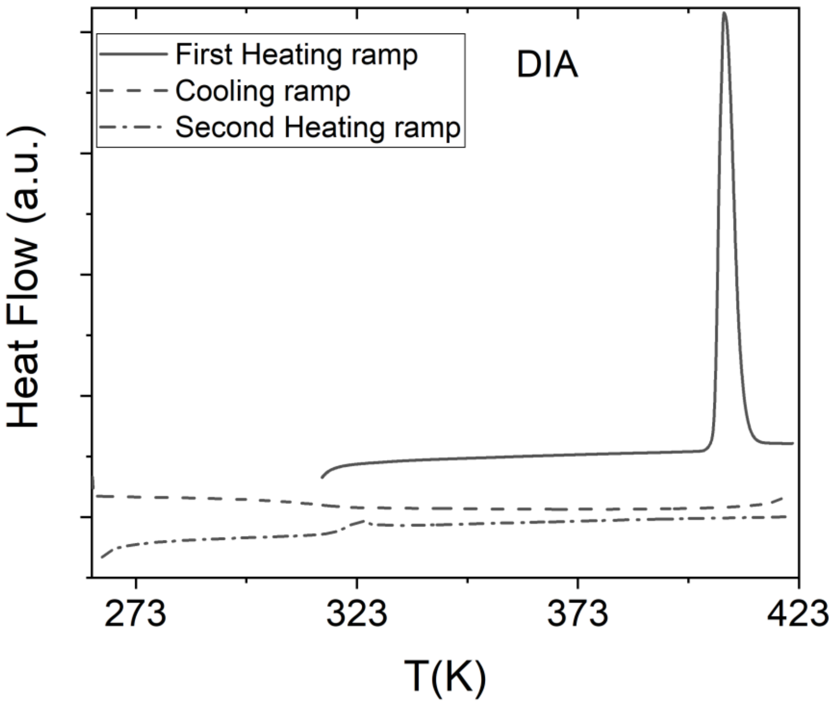

Figure S1. DSC traces of pure diazepam (DIA). Solid, dashed and dashed dotted lines represent the first heating ramp, the subsequent cooling and the second heating ramp, respectively. Endotherm transitions are represented upwards.

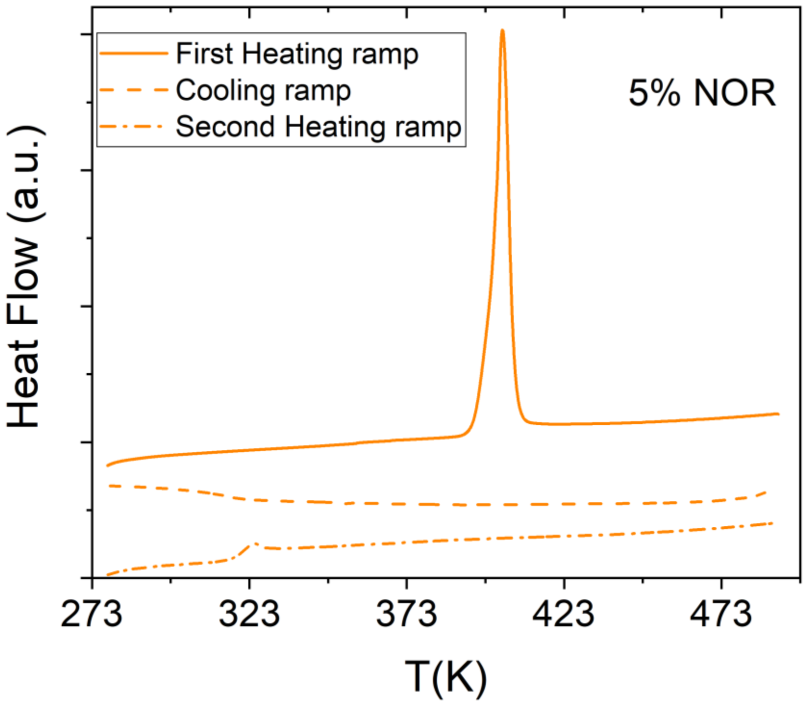

Figure S2. DSC traces of DIA with 5% nordazepam (NOR). Solid, dashed and dashed dotted lines represent the first heating ramp, the subsequent cooling and the second heating ramp, respectively. Endotherm transitions are represented upwards.

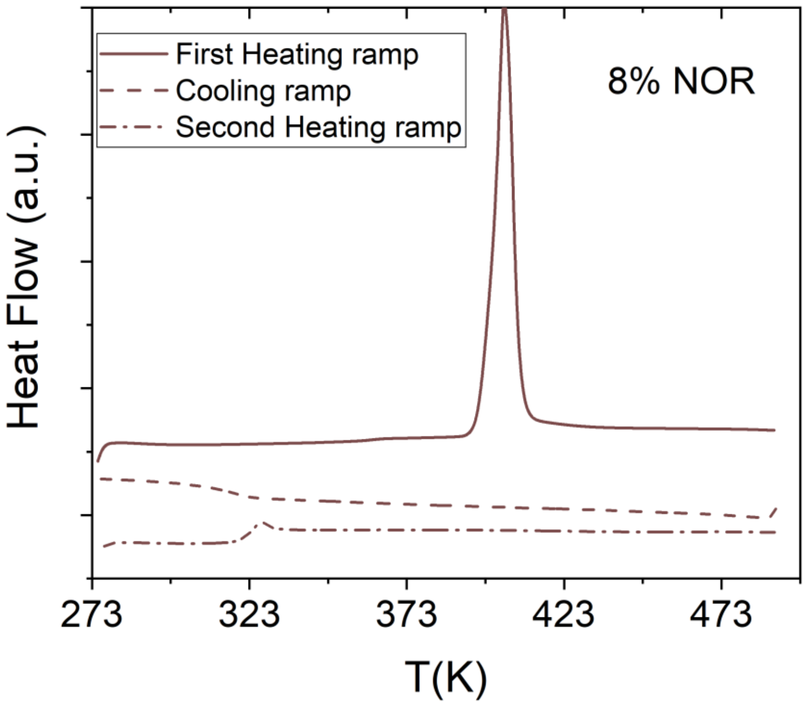

Figure S3. DSC traces of DIA with 8% NOR. Solid, dashed and dashed dotted lines represent the first heating ramp, the subsequent cooling and the second heating ramp, respectively. Endotherm transitions are represented upwards.

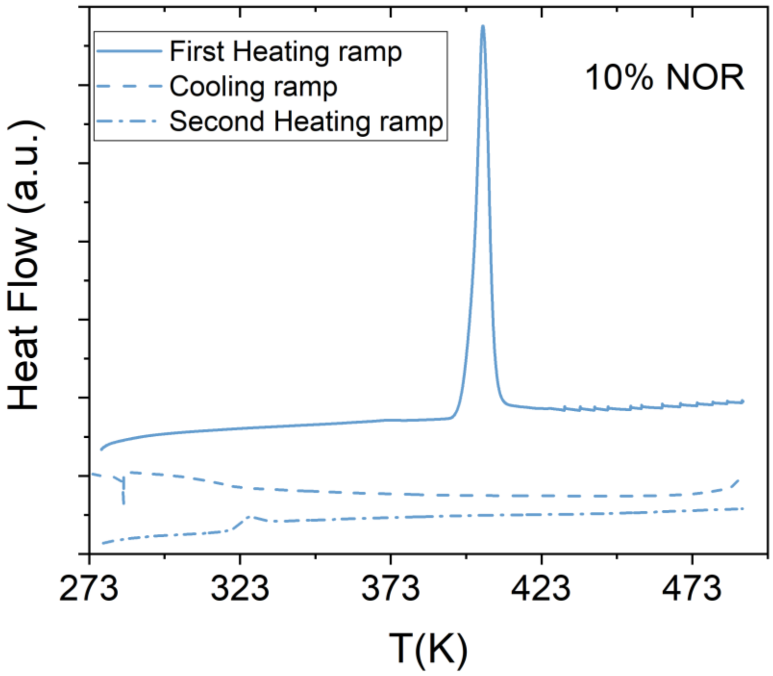

Figure S4. DSC traces of DIA with 10% NOR. Solid, dashed and dashed dotted lines represent the first heating ramp, the subsequent cooling and the second heating ramp, respectively. Endotherm transitions are represented upwards.

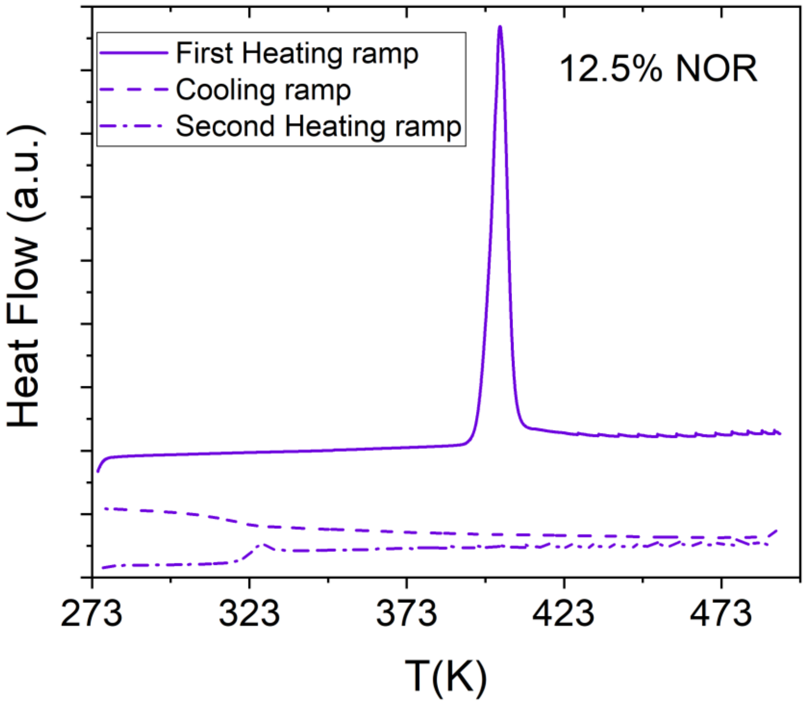

Figure S5. DSC traces of DIA with 12.5% NOR. Solid, dashed and dashed dotted lines represent the first heating ramp, the subsequent cooling and the second heating ramp, respectively. Endotherm transitions are represented upwards.

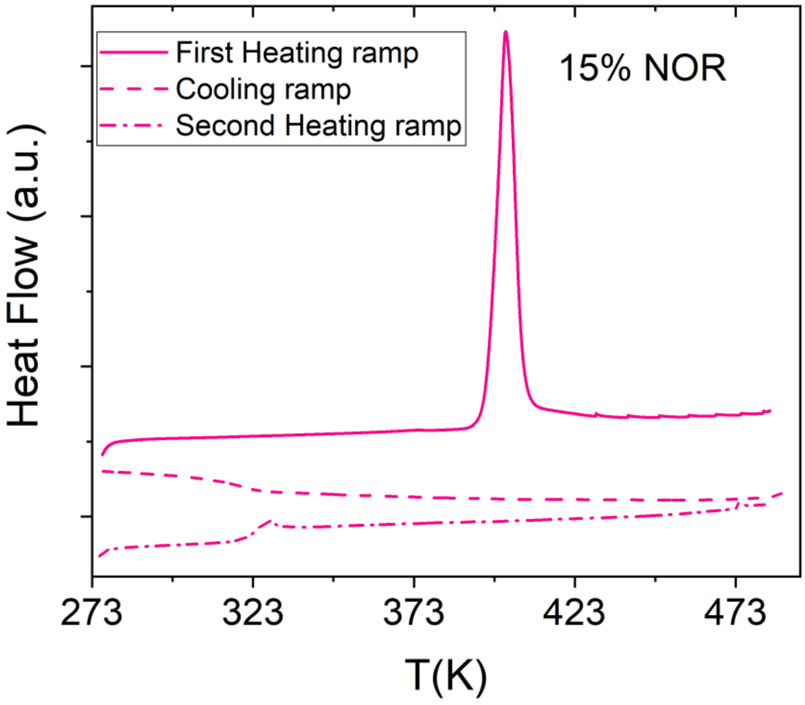

Figure S6. DSC traces of DIA with 15% NOR. Solid, dashed and dashed dotted lines represent the first heating ramp, the subsequent cooling and the second heating ramp, respectively. Endotherm transitions are represented upwards.

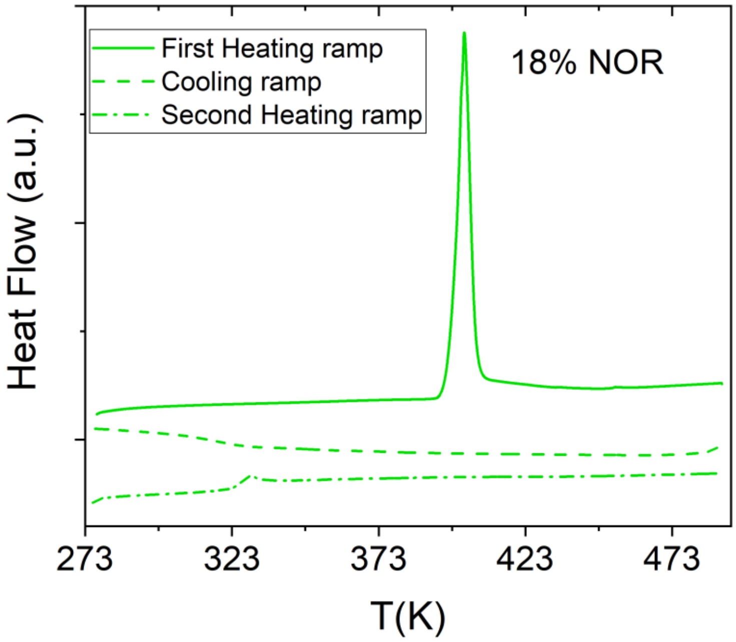

Figure S7. DSC traces of DIA with 18% NOR. Solid, dashed and dashed dotted lines represent the first heating ramp, the subsequent cooling and the second heating ramp, respectively. Endotherm transitions are represented upwards.

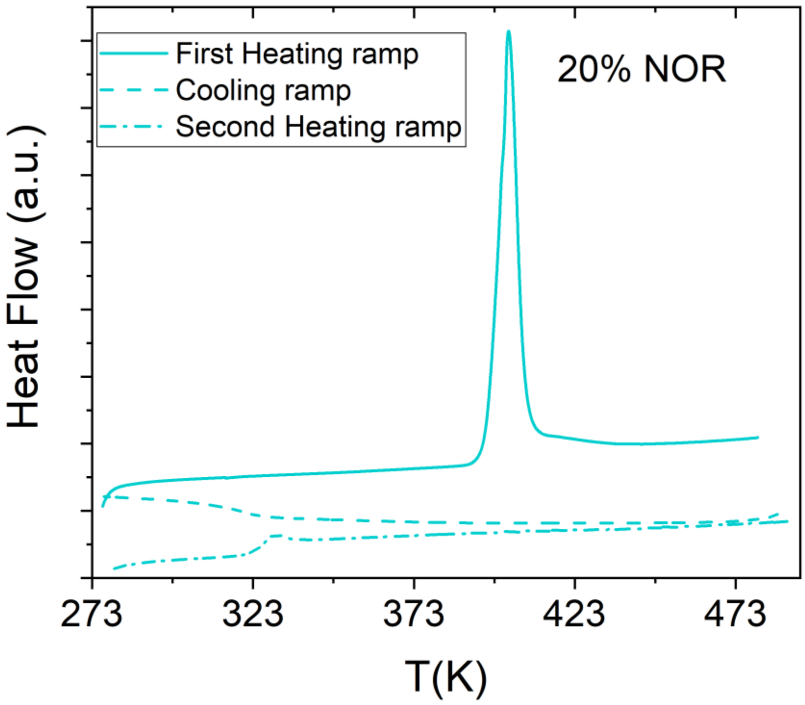

Figure S8. DSC traces of DIA with 20% NOR. Solid, dashed and dashed dotted lines represent the first heating ramp, the subsequent cooling and the second heating ramp, respectively. Endotherm transitions are represented upwards.

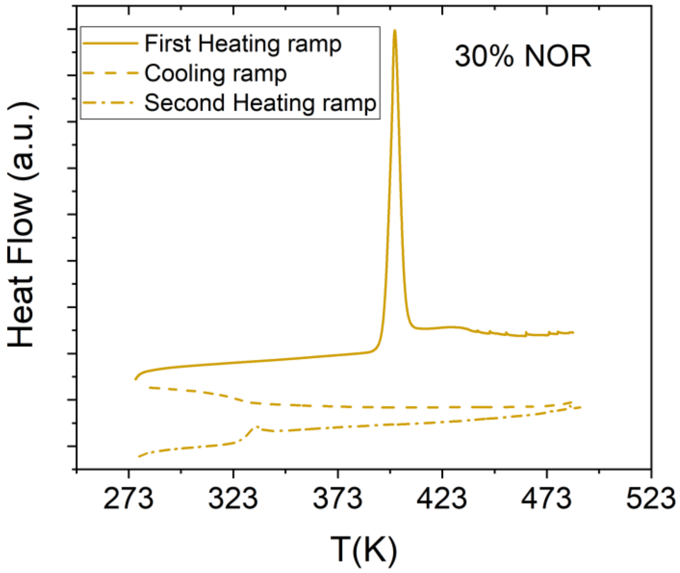

Figure S9. DSC traces of DIA with 30% NOR. Solid, dashed and dashed dotted lines represent the first heating ramp, the subsequent cooling and the second heating ramp, respectively. Endotherm transitions are represented upwards.

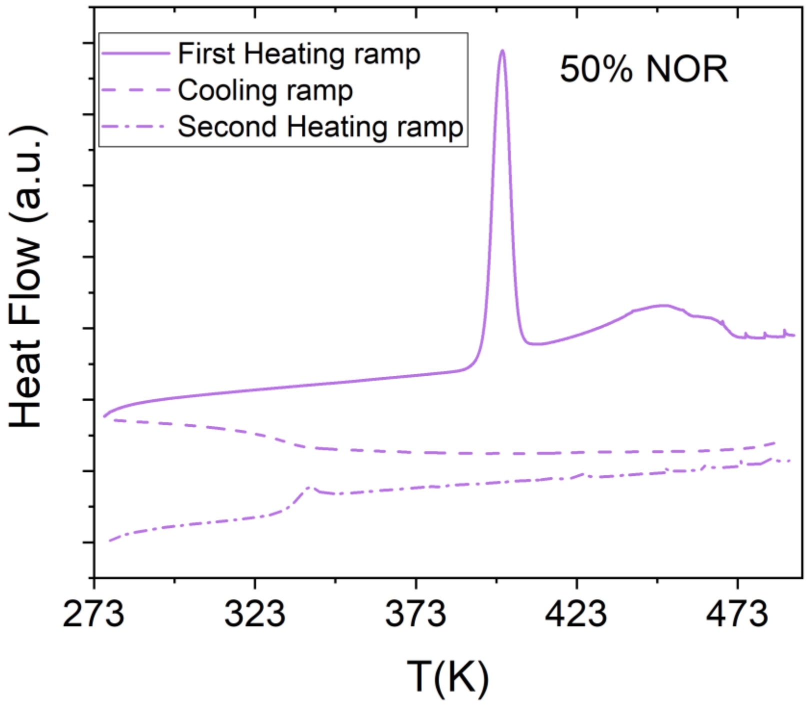

Figure S10. DSC traces of DIA with 50% NOR. Solid, dashed and dashed dotted lines represent the first heating ramp, the subsequent cooling and the second heating ramp, respectively. Endotherm transitions are represented upwards.

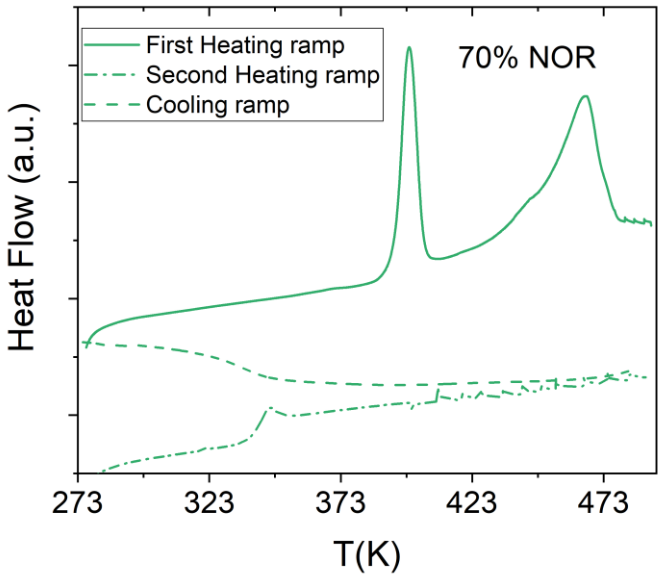

Figure S11. DSC traces of DIA with 70% NOR. Solid, dashed and dashed dotted lines represent the first heating ramp, the subsequent cooling and the second heating ramp, respectively. Endotherm transitions are represented upwards.

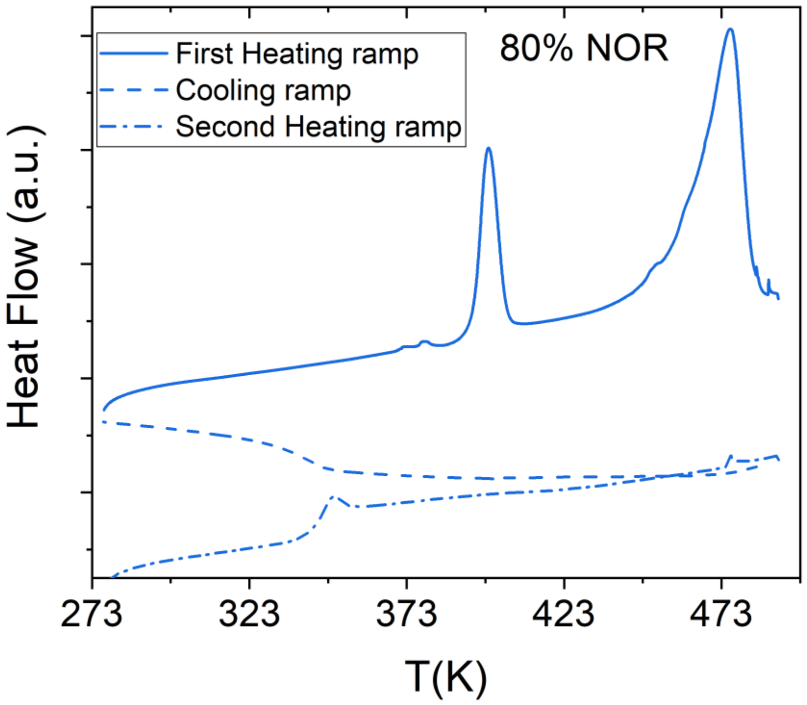

Figure S12. DSC traces of DIA with 80% NOR. Solid, dashed and dashed dotted lines represent the first heating ramp, the subsequent cooling and the second heating ramp, respectively. Endotherm transitions are represented upwards.

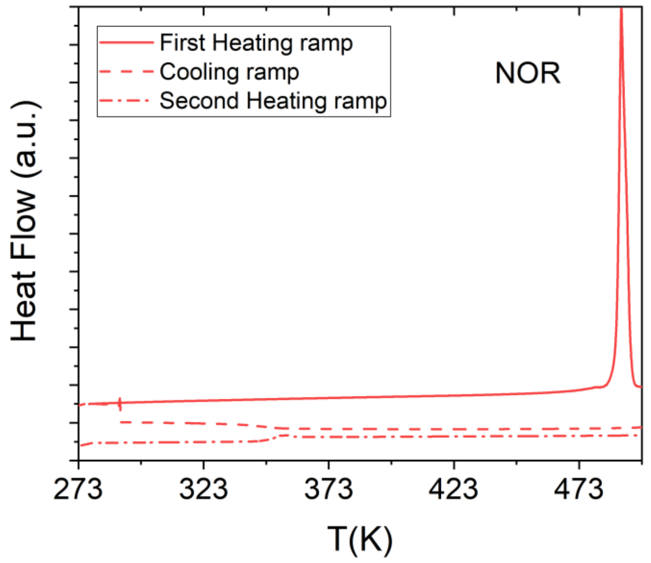

Figure S13. DSC traces of NOR. Solid, dashed and dashed dotted lines represent the first heating ramp, the subsequent cooling and the second heating ramp, respectively. Endotherm transitions are represented upwards.
